# The Association Between Higher Expression of Talin-1 and the Reduced Survival Rate in Ovarian Serous Carcinoma Patients

**DOI:** 10.30699/IJP.2023.554227.2901

**Published:** 2023-07-16

**Authors:** Mina Sharbatoghli, Leili Saeednejad Zanjani, Fattahi Fahimeh, Elham Kalantari, Zohre Habibi Shams, Mahshid Panahi, Mehdi Totonchi, Mohsen Asadi-Lari, Zahra Madjd

**Affiliations:** 1 *Oncopathology Research Center, Iran University of Medical Sciences (IUMS), Tehran, Iran*; 2 *Department of Embryology, Reproductive Biomedicine Research Center, Royan Institute for Reproductive Biomedicine, ACECR, Tehran, Iran*; 3 *Department of Pathology and Genomic Medicine, Sidney Kimmel Cancer Center, Thomas Jefferson University, Philadelphia, PA, USA*; 4 *Clinical Research Development Unit of Ayatollah-Khansari Hospital, Arak University of Medical Sciences, Arak, Iran*; 5 *Department of Pathology, Iran University of Medical Sciences, Tehran, Iran *; 6 *Department of Stem Cells and Developmental Biology, Cell Science Research Center, Royan Institute for Stem Cell Biology and Technology, ACECR, Tehran, Iran*; 7 *Department of Genetics, Reproductive Biomedicine Research Center, Royan Institute for Reproductive Biomedicine, ACECR, Tehran, Iran*; 8 *Department of Epidemiology, School of Public Health, Iran University of Medical Sciences, Tehran, Iran*; 9 *Department of Molecular Medicine, Faculty of Advanced Technologies in Medicine, Iran University of Medical Sciences, Tehran, Iran*; # * M Sharbatoghli and L Saeednejad Zanjani contributed equally to this work as the first author position.*

**Keywords:** Cancer progression, Ovarian serous carcinoma, Prognosis, Talin-1, Tissue microarray

## Abstract

**Background & Objective:**

Talin-1 is a constituent of the multiprotein adhesion complexes that play main role in the formation of tumors and migration in different types of malignancies. The present study aimed to assess expression and prognostic significance of the talin-1 protein in ovarian serous carcinoma (OSC) patients.

**Methods:**

The expression of talin-1 in mRNA and its protein levels were investigated for ovarian cancer (OC) by using bioinformatics tools, including Gene Expression Profiling Interactive Analysis 2 (GEPIA2), Gene Expression Database of Normal and Tumor Tissue 2 (GENT2), and The University of ALabama at Birmingham CANcer data analysis Portal (UALCAN) databases. Thereafter, immunohistochemical (IHC) staining was used to study the expression patterns of the talin-1 protein using 46 paraffin-embedded OSC tissue specimens, 25 benign tumors, and 20 normal tissues, which were assembled in tissue microarrays (TMAs). We also assessed the potential association between the expression of the talin-1 protein, various clinicopathological parameters, and survival outcomes.

**Results:**

Our IHC examination for talin-1 was significantly overexpressed in OSC tissues compared to benign tumors and normal tissues. The Kaplan-Meier survival analysis has also indicated statistically significant differences in terms of disease-specific survival (DSS) and progression-free survival (PFS) between the patients with high and low expression levels of talin-1, respectively.

**Conclusion:**

The talin-1 protein was overexpressed in OSC tissues, and a high expression level of talin-1 was found to be significantly associated with tumor aggressiveness and poorer DSS or PFS. Therefore, talin-1 may serve as a molecular marker of cancer progression and a novel prognostic biomarker in these patients.

## Introduction

Ovarian cancer (OC) is a lethal cancer of the female gynecologic organs, which is ranked as the fifth deadliest cancer among women aged between 15 and 75 years old. Moreover, it accounts for 1.3% of all new cancer cases and 2.3% of all cancer mortalities (1). The incidence of new OC cases is 11.2 per 100,000 women per year, and the mortality rate is 6.9 per 100,000 women per year (2).

The most common OC is epithelial OC (EOC), which accounts for approximately 85%-90% of all ovarian tumors (3). Ovarian serous carcinoma (OSC) is the most prevalent and aggressive type of EOC, with approximately 70% of cases detected at advanced stages (stage III or IV) (4). Several US Food and Drug Administration-approved serum tumor biomarkers have been used to screen high-risk OC in women. These include carcinoembryonic antigen (CEA), cancer antigen 125 (CA125), human epididymis protein 4 (HE4), risk of ovarian malignancy algorithm (ROMA), ova1, and overa (3). Notably, these markers are appropriate for diagnosis and have low accuracy and low sensitivity when used as prognostic biomarkers (5). In addition, the 5-year survival rate is less than 20% among OC cases (6); thus, identifying novel diagnostic and prognostic biomarkers of OC is urgently needed.

Talin-1 is known as one of the major components of focal adhesion. Inasmuch as talin-1 is a ubiquitous cytosolic protein that is able to link integrin to the actin cytoskeleton either directly or indirectly by interacting with vinculin and α-actinin (7). Additionally, the cytoplasmic domain of the integrin β subunit has an interaction with the talin head, leading to the activation and the enhanced binding of integrin to its ligand in the extracellular matrix (ECM) (8). Since talin-1 plays an important role in the interaction between integrin, cytoskeleton, and ECM, dysregulation of this protein has been linked to tumor metastasis and invasion (9). Studies conducted on OC have demonstrated that increased expression of the talin-1 protein is associated with the development of disease and progression to metastasis (10, 11). Accordingly, silencing the talin-1 gene could inhibit the invasion and migration of OC cells by preventing epithelial-mesenchymal transition (EMT) (11). Previous studies conducted on oral squamous cell carcinoma (OSCC) (12) and nasopharyngeal carcinoma (13) have shown that increased expression of the talin-1 protein leads to the progression of the disease and poor survival. A recent study performed by our group using talin-1 demonstrated that low expression of talin-1 on both mRNA and protein levels was significantly associated with advanced pathological features and worse disease-specific survival (DSS) in patients with colorectal cancer (CRC) (14). Moreover, the downregulation or the absence of talin-1 was found in hepatocellular carcinoma (HCC), endometriosis, and endometrioid carcinomas (15, 16). 

In the current study, in the initial step, comprehensive alterations in mRNA and protein levels of talin-1, which is known as a biomarker in OC patients, were analyzed using Gene Expression Profiling Interactive Analysis 2 (GEPIA2), Gene Expression Database of Normal and Tumor Tissue 2 (GENT2), and UALCAN databases. Our literature review showed conflicting results regarding the expression of talin-1 in OC patients. Therefore, the present study was designed to evaluate the protein expression of talin-1 in OSC patients. For this purpose, a series of formalin-fixed paraffin-embedded (FFPE) tissues obtained from OSC patients, benign ovarian tumors, and normal ovaries were investigated using the tissue microarrays (TMAs) technique. Thereafter, the associations among the expression levels of the talin-1 protein, clinicopathological parameters, and survival outcomes in the OSC group were analyzed.

## Material and Methods

**Investigating Talin-1 Expression in OC Patients Using Bioinformatics Tools**

To determine the expression of talin-1 in OC patients, GEPIA2 and GENT2 were used as user-friendly search online platforms for gene expression patterns. Correspondingly, GEPIA2 uses RNA sequencing data of both tumor and normal samples obtained from Cancer Genome Atlas (TCGA) and Genotype-Tissue Expression (GTEx) databases (17). While GENT2 includes gene expression data on cancer and normal tissues from the gene expression omnibus (GEO) on the National Center for Biotechnology Information (NCBI) database with 2 microarray platforms (namely Affymetrix U133A or U133Plus2) (18). Furthermore, GEPIA2 and GENT2 were used to evaluate the prognostic potential with the overall survival curves in the OC samples based on the median expression of talin-1. Moreover, the UALCAN database was used to compare the protein expression levels of talin-1 between the OC and normal tissue samples. UALCAN is a web-based tool providing mRNA and differential protein expression analysis for several types of cancer (19).

**Patients and Sample Collection**

The sample size of the study was computed using the information obtained on the main outcomes of the study. The intensity of staining in the 2 groups (group 1 [negative and weak] and group 2 [moderate and strong]): 27% in benign ovarian tumors and 0% in normal tissue samples. Taking into account the power of 0.8, a confidence level of 0.95, and a 2-sided test, we estimated a sample size of at least 22 samples per group using the PASS 2023 software ( NCSS, LLC, Kaysville, Utah, USA) (20). Therefore, a total of 46 paraffin-embedded tissue specimens from OSC tumors were collected from Firozgar Hospital, a referral university-based general hospital in Tehran, Iran, from 2011 to 2018. All the samples were collected from 1 center in the same time period as randomization. In addition, the patients had no history of chemotherapy or radiation therapy. Hematoxylin and eosin (H&E) stained slides and medical records were used to obtain clinicopathological characteristics, including age, tumor size (maximum tumor diameter), FIGO (Federation International of Gynecology and Obstetrics) stage, histological grade, lymph node metastasis, vascular invasion, omentum involvement, fallopian tube involvement, cervix involvement, endometrium involvement, myometrium involvement, vagina involvement, peritoneum involvement, lymphovascular space invasion, perineural invasion, colon involvement, small intestine involvement, post-cul-de-sac involvement, paracolic lymph node involvement, tumor recurrence, and distant metastasis. Furthermore, 25 benign tumor samples (including 15 serous cystadenomas and 10 mucinous cystadenomas) and 20 normal tissue samples were included to compare the expression levels of talin-1 in a wide range of tissue specimens. DSS was explained from the time of oophorectomy up to the date of death caused by cancer. Progression-free survival (PFS) was described as the length of time between the first surgery and the latest follow-up visit when the patient showed no detectable disease, metastasis, or recurrence. The stage and grade of OC were considered according to the FIGO Cancer Report 2018 and the World Health Organization (WHO) classification of ovarian tumors 2020 (21, 22). 

**TMA Structure**

The H&E slides were used to select and label the 3 most representative zones of tumor tissues by an expert pathologist (M. P.). Each paraffin-embedded donor block, as representative of tumor areas, was transferred into TMA recipient blocks in 3 copies using the TMA equipment (Minicore, Alphelys, France) as previously described (23, 24). The sections were prepared for immunohistochemical (IHC) staining after cutting the recipient blocks and transferring them onto adhesive slides (4 mm).

In a TMA study, 1 core represented more than 90% of the expression pattern of the whole tissue. While the analyses of 2 cores revealed between 95% and 99% accuracy and validity of the antigen expression pattern (25). In this study, we evaluated 3 copies of each sample to achieve greater accuracy and validity of the analysis, besides overcoming heterogeneity of antigen expression patterns.

**Immunohistochemistry**

The IHC staining method was performed based on a standard chain polymer-conjugated (Envision) technique as described earlier (26, 27). In brief, all TMA sections were dewaxed, rehydrated by xylene, and then graded by ethanol treatment. Subsequently, 3% H_2_O_2_ was used to block endogenous peroxides for 20 min at 25 °C. After the washing procedure, antigen retrieval was done by autoclaved slides immersed in citrate buffer (pH=6.0) for 10 min. Thereafter, TMA slides were incubated with anti–talin-1 antibody (ab71333, Abcam, Cambridge, MA, USA) and diluted 1/1000 as the primary antibody overnight at 4°C. Moreover, rabbit immunoglobulin G (Invitrogen, Thermo Fisher Scientific, Waltham, MA, USA) was used as the isotype control to confirm the nonspecific bindings of the primary antibody. Next, the tissue sections were incubated with a secondary antibody (anti-mouse/rabbit HRP polymer [EUROMAB, UMR1000PD, USA]) for 40 min. Moreover, TMA slides were exposed to 3, -3'diaminobenzidine substrate (DAB, Dako, Glostrup, Denmark) as chromogen for 5 min at 25°C, and the slides were then counterstained with hematoxylin (Dako, Glostrup, Denmark) to visualize the antigen. The tissue sections were finally dehydrated in alcohol and cleared with xylene. In this study, normal human kidney tissue was used as a positive control, while Tris-buffered saline (TBS) was used as a negative control instead of the primary antibody to validate the nonspecific bindings of a secondary antibody.

**IHC Scoring System**

The IHC staining of OC TMAs was independently assessed by 2 pathologists (M. P. and M. S.) blinded to the patient’s pathological information. An agreement of scoring was received for all the specimens. The semi-quantitative system was applied to score the intensity of staining, which was arranged from negative to strong as follows: 0, negative; 1, weak; 2, moderate; and 3, strong. The percentages of positive cells were categorized according to the positive tumor cells from 1% to 100% as follows: less than 25% as positive cells, between 25% and 50% as positive cells, between 51% and 75% as positive cells, and more than 75% as positive cells. Notably, the histochemical score (H score) was achieved by multiplying the intensity score by the percentage of positive cells, which yielded a range from 0 to 300. The median of the H score (H score=100) was used as a cutoff point to categorize the tumors with high or low talin-1 expression.

**Statistics**

SPSS version 22 (SPSS Inc., Chicago, IL., USA) was used to analyze the obtained data. We have described the categorical data by N (%), a valid percentage, and quantitative data, including mean (SD) and median (Q1, Q3). Kruskal-Wallis and Mann-Whitney *U* tests were run for pairwise comparison between the groups. In addition, Pearson’s chi-square and Spearman’s correlation tests were used to analyze the significance of the association and correlation between the expression of the talin-1 protein and clinicopathological features. Furthermore, DSS and PFS curves were drawn by adopting the Kaplan-Meier method, and the log-rank test was also performed to compare the estimated curves between the groups with 95% CI. Cox regression modeling was finally performed for all variables to determine which variables affected DSS or PFS. As a result, those variables that significantly affected survival in univariate analysis were included in multivariable analyses. P-values less than 0.05 were considered statistically significant.

## Results

**Bioinformatics Approach Applied to the Expression of Talin-1 in OC Patients**

**Fig. 1:**
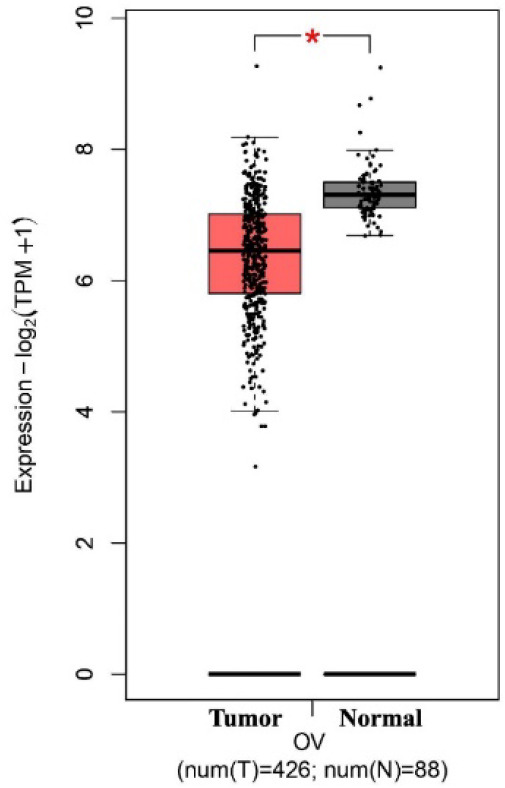
The mRNA expression level of Talin-1 in the ovarian serous carcinoma (OSC) using Gene Expression Profiling Interactive Analysis2 (GEPIA2).

**Fig. 2:**
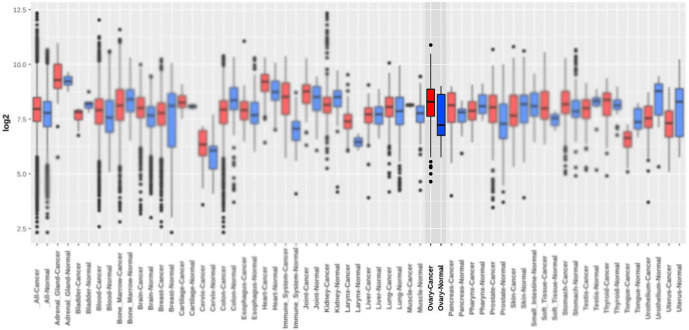
The mRNA expression level of Talin-1 in the ovarian cancer (OC) using Gene Expression database of Normal and the Tumor tissues 2 (GENT2).

**Fig. 3:**
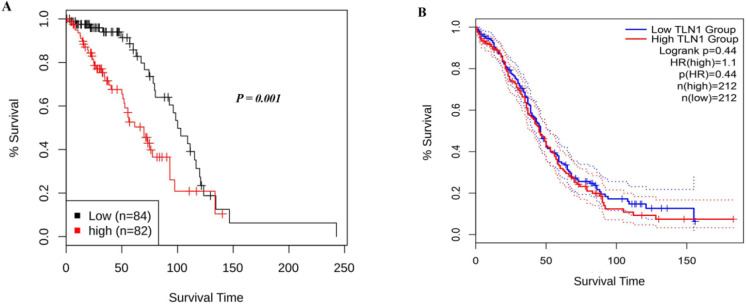
Kaplan-Meier survival curves analysis for overall survival (OS) of Talin-1 mRNA expression in the OC patients using bioinformatics tools. A) High Talin-1 expression was found to be associated with a poor prognosis in the OC patients using gene expression database on both normal and the tumor tissues 2 (GENT2) B). The results revealed that OS analysis was not statistically significant using the Gene Expression Profiling Interactive Analysis (GEPIA2) tool.

**Fig. 4:**
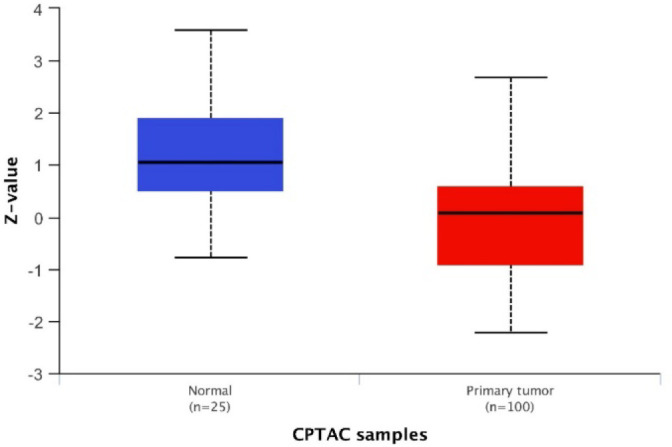
Box plot of the clinical proteomic tumor analysis consortium (CPTAC) results for Talin-1 expression on UALCAN database.

The results of the TCGA database using the GEPIA2 tool revealed that the mRNA expression level of talin-1 was significantly lower in the OC tissues than in the normal tissues (Log_2_FC=0.5 and *P*<0.05; Figure 1). However, the mRNA expression level of talin-1 was significantly higher in the OC tissues than in the normal tissues based on GENT2 (GPL 96 platform), as shown in Figure 2 and Table 1. Next, the prognostic value of talin-1 was analyzed by GEPIA2 and GENT2 databases using transcriptomic data on OC patients. As a result, a high expression level of talin-1 was found to be associated with worse OS in OC tissues through GENT2 (*P*=0.001); however, the result obtained from the GEPIA2 was not significant (*P*=0.44; Figures 3A and 3B). Additionally, the results of the UALCAN database demonstrated that the expression of talin-1 was significantly reduced in the primary tumor of OC compared to the normal tissue samples (*P*<0.0001; Figure 4). 

**Table 1:** Investigation of Talin-1 on the GENT2 database

Microarray platforms	P-value	Log_2_FC
GPL96platform (HG-U133A)]	0.005	0.681
GPL570 platform (HG-U133_Plus_2)	0.363	0.119

**Table 2:** Expression of Talin-1 (Intensity of staining, percentage of positive tumor cells, and H-score) in the ovarian serous carcinoma (OSC), benign ovarian tumors, and normal tissue samples.

Scoring system	Ovarian cancer N (%)	Benign ovarian tumors N (%)	Normal tissue samples N (%)	*P*
Intensity of staining Negative (0) Weak (+1) Moderate (+2) Strong (+3)	8 (17.4) 19 (41.3) 11 (23.9) 8 (17.4)	7 (28.0) 12 (48.0) 6 (24.0) 0 (0.0)	11 (55.0) 9 (45.0) 0 (0.0) 0 (0.0)	***0.004***
Percentage of positive tumor cells <25% 25–50% 51- 75% > 75%	8 (17.4) 0 (0.0) 0 (0.0) 38 (82.6)	7 (28.0) 0 (0.0) 0 (0.0) 18 (72.0)	12 (60.0) 2 (10.0) 3 (15.0) 3 (15.0)	0.301
H-score cut off =100 Low High	27 (58.7) 19 (41.3)	25 (100.0) 0 (0.0)	12 (60.0) 8 (40.0)	***0.048***
Total	46	25	20	

*H-score* histological score

P-values are based on Pearson’s χ2 test

**Characteristics of OSC Patients**

Forty-six paraffin-embedded OSC tissue samples were included in this study. The mean age of the patients was 45 (SD=14.8) years old (ranging from 16 to 74); 18 (39.1%) patients were younger than 45 years old, and 28 (60.9%) subjects were over 45 years old. The range of tumor size at the largest diameter was found to be between 1 and 23 cm, and tumors were classified into the following 2 groups based on the mean of tumor size (8 cm): group 1 (more than 8 cm or equal to 8 cm; n=25 [54.3%]) and group 2 (less than 8 cm; n=11 [23.9%]). We had no information on the tumor size in 10 patients (21.7%). In this study, 24 (53.3%) tumors had low histological grade, and 21 (46.7%) cases had high histological grade. Moreover, 10 (21.7%) cases were in FIGO stage I, 15 (32.6%) were in FIGO stage II, and 21 (45.7%) were in FIGO stage III. Lymph node and hematogenous metastases were found in 19 (41.3%) and 6 (13.0%) cases, respectively. Furthermore, tumor recurrence and distant metastasis were observed in 26 (56.5%) and 21 (45.7%) patients, respectively, during the follow-up period. Other sites of involvement were as follows: omentum in 9 (19.6%) cases, fallopian tube in 10 (21.7%) cases, cervix in 9 (19.6%) cases, endometrium in 4 (8.7%) cases, myometrium in 6 (13.0%) cases, vagina in 11 (23.9%) cases, peritoneum in 4 (8.7%) cases, colon in 9 (19.6%) cases, small intestine in 4 (8.7%) cases, post-cul-de-sac in 8 (17.4%) cases, paracolic lymph node in 4 (8.7%) cases, lymphovascular space invasion in 7 (15.2%) cases, and perineural invasion in 4 (8.7%) cases.

**Expression of Talin-1 in OSC, Benign Tumors, and Normal Tissue Samples**

**Fig. 5:**
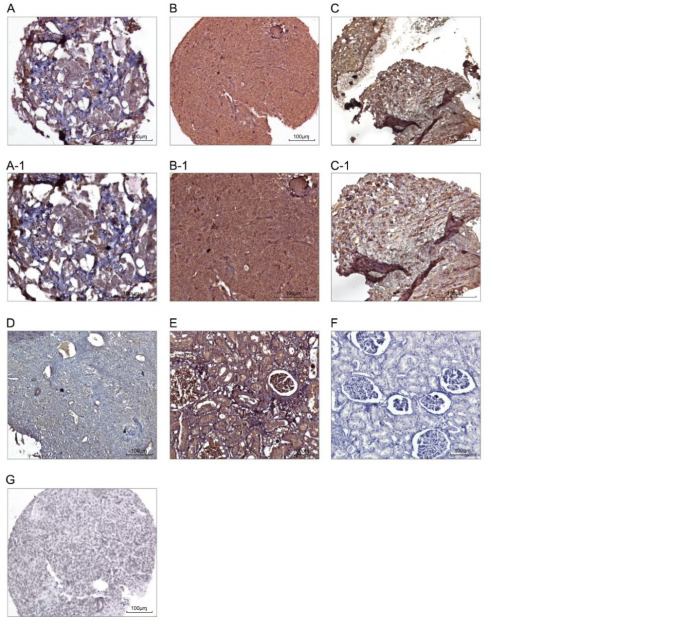
Immunohistochemical staining of Talin-1 protein in the ovarian serous carcinoma (OSC) patients, benign tumors, and normal tissues. Low cytoplasmic expression of Talin-1 (A-A-1) and high cytoplasmic Talin-1 expression (B-B-1). Expression of Talin-1 in the benign tumors (C-C-1). Expression of Talin-1 in normal tissues (D). Human normal kidney tissues as controls: positive (E) and negative (F). Isotype control (G). The expression level of Talin-1 was significantly higher in theOSC tissues compared to benign tumors and normal tissue samples. Figures have magnification of 100 × and 200×.

To examine the expression pattern and clinical significance of talin-1, its expression level was analyzed in a set of 46 paraffin-embedded OSC tissue samples using IHC technique on TMA slides and by applying 3 scoring methods, including intensity of staining, percentage of positive tumor cells, and H score (Table 2). The expression level of talin-1 was also evaluated in benign tumors and normal tissue samples. Positive staining of talin-1 was mainly observed in the cytoplasm of the tissue samples. It is noteworthy that the level of expression of talin-1 was significantly higher in the OSC tissues compared to the benign tumors and normal tissue samples. The mean levels of expression of talin-1 (H score) in OSC, benign tumors, and normal tissue samples were 142, 96, and 35, respectively (*P*<0.05; Figure 5).

**Associations Between Expression of Talin-1 and the Clinicopathological Characteristics**

In the current study, Pearson’s chi-square test was used to examine the association between talin-1 expression and the clinicopathological characteristics of OSC. As a result, a statistically significant difference was found between the expression of talin-1 and the high histological grade in terms of intensity and H score (Pearson's Chi-square; *P*=0.001 and *P*<0.001, respectively), advanced FIGO stage (H score; *P*=0.017), and myometrium involvement (H score; *P*=0.037; Table 3). Moreover, the results of Spearman’s correlation analysis showed a significant direct correlation between the expression of talin-1 and high histological grades in terms of intensity and H score (Spearman’s rho; *P*=0.002 and *P*<0.001), advanced FIGO stage (*P*=0.004 and *P*=0.004), and myometrium involvement (*P*=0.049 and *P*=0.038).

 In the present research, the nonparametric Kruskal-Wallis and Mann-Whitney *U* tests were also used to compare the differences between the median expressions of talin-1 between different groups. The Kruskal-Wallis test showed a statistically significant difference in the median levels of talin-1 expression in various histological grades and FIGO stages (*P*=0.004 and *P*=0.018). Moreover, the Mann-Whitney *U* test indicated a statistically significant difference between the median expression levels of talin-1 in low- (*P*<0.001) and high-grade tumors (*P*<0.001). Furthermore, a statistically significant difference was found between the median expression levels of talin-1 in FIGO stages I and III (*P*=0.011), as well as between stages II and III (*P*=0.039). However, no statistically significant differences were observed between the median expression levels of talin-1 in histological grades and FIGO stages I and II (*P*=0.477 and *P*=0.545). In addition, we found no significant association between the expression levels of talin-1 and other clinicopathological characteristics (Table 3). 

**Table 3:** Association between expression of Talin-1 and the clinicopathological parameters of the ovarian serous carcinoma (OSC) samples (Intensity of staining and H-score) (*P* *value*; Pearson’s χ2 test)

Patients and tumor characteristics	Total samples N (%)	Intensity of staining N (%)							*P*	H-score (cut off = 100) N (%)		*P*
		0 (Negative)	1+ (Weak)			2+ (Moderate)	3+ (Strong)			Low (≤100)	High (>100)	
Mean age, years (Range)	45 (16-74)											
≤Mean age	18 (39.1)	3 (37.5)		7 (36.8)	4 (36.4)			4 (50.0)	0.923	10 (37.0)	8 (42.1)	0.729
>Mean age	28 (60.9)	5 (62.5)		12 (63.2)	7 (63.6)			4 (50.0)		17 (63.0)	11 (57.9)	
Mean tumor size (cm)	8.0											
≤Mean	25 (54.3)	5 (62.5)		9 (75.5)	5 (50.0)			6 (100.0)	0.189	14 (70.0)	11 (68.8)	0.936
>Mean	11 (23.9)	3 (37.5)		3 (25.0)	5 (50.0)			0 (0.0)		6 (30.0)	5 (31.3)	
Not identified	10 (21.7)	0 (0.0)		0 (0.0)	0 (0.0)							
Histological grade												
Low grade	24 (52.2)	4 (50.0)		17 (89.5)	3 (27.3)			0 (0.0)	***< 0.001***	21 (77.8)	6 (22.2)	***< 0.001***
High grade	22 (47.8)	4 (50.0)		2 (10.5)	8 (72.7)			8 (100.0)		3 (15.8)	16 (84.2)	
FIGO stage												
I	10 (21.7)	2 (20.0)		7 (70.0)	1 (10.0)			0 (0.0)		9 (33.3)	1 (5.3)	
II	15 (32.6)	4 (26.7)		6 (31.6)	4 (36.4)			1 (12.5)	0.082	10 (37.0)	5 (26.3)	***0.017***
III	21 (45.7)	2 (25.0)		6 (31.6)	6 (54.5)			7 (87.5)		8 (29.6)	13 (68.4)	
IV	0 (0.0)	0 (0.0)		0 (0.0)	0 (0.0)			0 (0.0)		0 (0.0)	0 (0.0)	
Lymph node (LN) metastasis												
Involved	19 (41.3)	1 (12.5)		7 (43.8)	7 (63.6)			4 (50.0)		8 (33.3)	11 (57.9)	
None	24 (52.2)	7 (87.5)		9 (56.3)	4 (36.4)			4 (50.0)	0.168	16 (66.7)	8 (42.1)	0.107
Not identified	3 (6.5)	0 (0.0)		0 (0.0)	0 (0.0)			0 (0.0)				
Vascular invasion (VI)												
Involved	6 (13.0)	1 (12.5)		1 (6.3)	2 (18.2)			2 (25.0)	0.619	2 (8.3)	4 (21.1)	0.232
None	37 (80.4)	7 (87.5)		15 (93.8)	9 (81.8)			6 (75.0)		22 (91.7)	15 (78.9)	
Not identified	3 (6.5)	0 (0.0)		0 (0.0)	0 (0.0)			0 (0.0)				
Omentum Involved	9 (19.6)	2 (25.0)		1 (6.3)	2 (18.2)			4 (50.0)		3 (12.5)	6 (31.6)	
Omentum None	34 (73.9)	6 (75.0)		15 (93.8)	9 (81.8)			4 (50.0)	0.098	21 (87.5)	13 (68.4)	0.127
Not identified	3 (6.5)	0 (0.0)		0 (0.0)	0 (0.0)			0 (0.0)				
Fallopian tube Involved	10 (21.7)	2 (25.0)		1 (6.3)	4 (36.4)			3 (37.5)		3 (12.5)	7 (36.8)	
Fallopian tube None	33 (71.7)	6 (75.0)		15 (93.8)	7 (63.6)			5 (62.5)	0.206	21 (87.5)	12 (63.2)	0.061
Not identified	3 (6.5)	0 (0.0)		0 (0.0)	0 (0.0)			0 (0.0)				
Cervix Involved	9 (19.6)	2 (25.0)		2 (12.5)	2 (18.2)			3 (37.5)		4 (16.7)	5 (26.3)	
Cervix None	34 (73.9)	6 (75.0)		14 (87.5)	9 (81.8)			5 (62.5)	0.543	20 (83.3)	14 (73.7)	0.440
Not identified	3 (6.5)	0 (0.0)		0 (0.0)	0 (0.0)			0 (0.0)				
Endometrium Involved	4 (8.7)	0 (0.0)		2 (12.5)	2 (18.2)			0 (0.0)		2 (8.3)	2 (10.5)	
Endometrium None	39 (84.8)	8 (100.0)		14 (87.5)	9 (81.8)			8 (100.0)	0.413	22 (91.7)	17 (89.5)	0.806
Not identified	3 (6.5)	0 (0.0)		0 (0.0)	0 (0.0)			0 (0.0)				
Myometrium Involved	6 (13.0)	0 (0.0)		1 (6.3)	3 (27.3)			2 (25.0)		1 (4.2)	5 (26.3)	
Myometrium None	37 (80.4)	8 (100.0)		15 (93.8)	8 (72.7)			6 (75.0)	0.210	23 (95.8)	14 (73.7)	***0.037***
Not identified	3 (6.5)	0 (0.0)		0 (0.0)	0 (0.0)			0 (0.0)				
Vagina Involved	11 (23.9)	2 (25.0)		3 (18.8)	2 (18.2)			4 (50.0)		5 (20.8)	6 (31.6)	
Vagina None	32 (69.6)	6 (75.0)		13 (81.3)	9 (81.8)			4 (50.0)	0.360	19 (79.2)	13 (68.4)	0.423
Not identified	3 (6.5)	0 (0.0)		0 (0.0)	0 (0.0)			0 (0.0)				
Peritoneum Involved	4 (8.7)	1 (12.5)		1 (5.9)	0 (0.0)			2 (25.0)		2 (8.0)	2 (10.5)	
Peritoneum None	40 (87.0)	7(87.5)		16 (94.1)	11 (100.0)			6 (75.0)	0.275	23 (92.0)	17 (89.5)	0.773
Not identified	2 (4.3)	0 (0.0)		0 (0.0)	0 (0.0)			0 (0.0)				
Lymphovascular space invasion												
Involved	7 (15.2)	2 (25.0)		1 (5.9)	2 (18.2)			2 (25.0)		3 (12.0)	4 (21.1)	
None	37 (80.4)	6 (75.0)		16 (94.1)	9 (81.8)			6 (75.0)	0.511	22 (88.0)	15 (78.9)	0.416
Not identified	2 (4.3)	0 (0.0)		0 (0.0)	0 (0.0)			0 (0.0)				
Perineural invasion												
Present	4 (8.7)	2 (25.0)		0 (0.0)	0 (0.0)			2 (25.0)	0.059	2 (8.3)	2 (10.5)	
Absent	39 (84.8)	6 (75.0)		16 (100.0)	11 (100.0)			6 (75.0)		22 (91.7)	17 (89.5)	0.806
Not identified	2 (4.3)	0 (0.0)		0 (0.0)	0 (0.0)			0 (0.0)				
Colon												
Involved	9 (19.6)	1 (12.5)		2 (11.8)	3 (27.3)			3 (37.5)	0.416	3 (12.0)	6 (31.6)	
None	35 (76.1)	7 (87.5)		15 (88.2)	8 (72.7)			5 (62.5)		22 (88.0)	13 (68.4)	0.111
Not identified	2 (4.3)	0 (0.0)		0 (0.0)	0 (0.0)			0 (0.0)				
Small intestine Involved	4 (8.7)	1 (12.5)		0 (0.0)	1 (9.1)			2 (25.0)		1 (4.2)	3 (15.8)	
None	39 (84.8)	7 (87.5)		16 (100.0)	10 (90.9)			6 (75.0)	0.253	23 (95.8)	16 (84.2)	0.193
Not identified	3 (6.5)	0 (0.0)		0 (0.0)	0 (0.0)			0 (0.0)				
Post-cul-de-sac Involved	8 (17.4)	2 (25.0)		2 (12.5)	1 (9.1)			3 (37.5)		4 (16.7)	4 (21.1)	
None	35 (76.1)	6 (75.0)		14 (87.5)	10 (90.9)			5 (62.5)	0.369	20 (83.3)	15 (78.9)	0.714
Not identified	3 (6.5)	0 (0.0)		0 (0.0)	0 (0.0)			0 (0.0)				
Paracolic lymph node												
Involved	4 (8.7)	1 (12.5)		3 (18.8)	0 (0.0)			0 (0.0)		4 (16.7)	0 (0.0)	
None	39 (84.8)	7 (87.5)		13 (81.3)	11 (100.0)			8 (100.0)	0.291	20 (83.3)	19 (100.0)	0.062
Not identified	3 (6.5)	0 (0.0)		0 (0.0)	0 (0.0)			0 (0.0)				
Distant metastasis Present	21 (45.7)	3 (37.5)		7 (36.8)	5 (45.5)			6 (75.0)	0.310	10 (37.0)	11 (57.9)	0.162
Absent	25 (54.3)	5 (62.5)		12 (63.2)	6 (54.5)			2 (25.0)		17 (63.0)	8 (42.1)	
Tumor recurrence												
Yes No	26 (56.5) 29 (43.5)	4 (50.0) 4 (100.0)		9 (47.4) 10 (52.6)	6 (54.5) 5 (45.5)			7 (87.5) 1 (12.5)	0.269	13 (48.1) 14 (51.9)	13 (68.4) 6 (31.6)	0.172

*H-score* histological score

Values in bold are statistically signiﬁcant

**Information on the Clinical Outcomes in the OSC Patients**

Our study revealed that the mean and median follow-up durations for DSS were (33; SD = 18.9 and 24; Q1, Q3= 22, 43) months, while those for PFS were (26; SD = 19.8 and 24; Q1, Q3= 12, 31), months. The minimum, maximum, and range of these follow-up times were 5, 84, and 79 months for DSS and 1, 84, and 83 months for PFS, respectively. During the follow-up period, cancer-related deaths were observed in 18 patients (39.1%). Our findings showed that tumor recurrence and distant metastasis occurred in 26 (56.5%) and 21 (45.7%) cases, while 20 (43.5%) and 25 (54.3%) subjects were shown as negative for the above-mentioned parameters, respectively. Additionally, 27 (58.7%) patients were positive for both tumor recurrence and distant metastasis, but 19 (41.3%) patients were negative for these 2 features. 

**Survival** **Analysis** **(DSS or PFS) Based on the Expression of Talin-1 **

Kaplan-Meier survival analysis was utilized to estimate DSS and PFS in relation to the expression of Talin-1 (H-score) in OSC tissue specimens. 

**Fig. 6:**
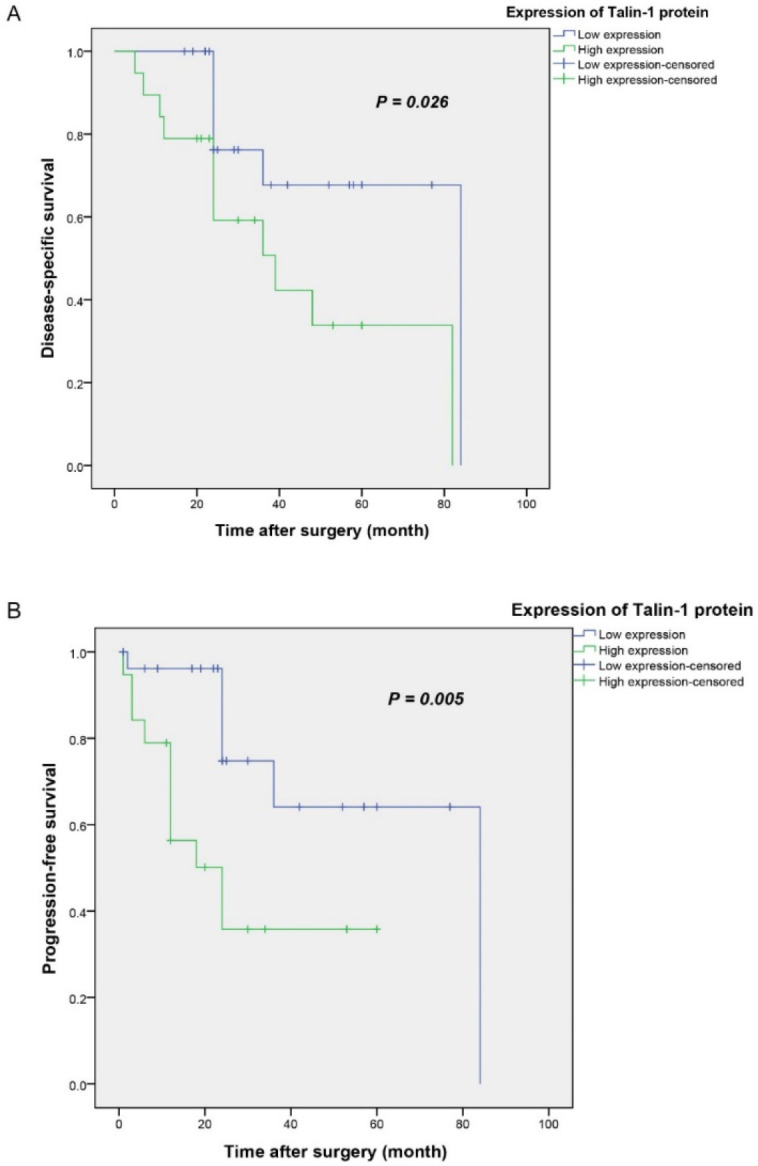
Kaplan–Meier curves for disease-specific survival (DSS) and progression-free survival (PFS) according to the expression levels of Talin-1 protein in the ovarian serous carcinoma (OSC) patients. A higher level of Talin-1 protein expression was found to be associated with shorter DSS (A) and PFS (B) compared to the tumors with low Talin-1 protein expression (*P*=0.026, *P*= 0.005, respectively).

The mean DSS/PFS time for the patients with high and low expression levels of talin-1 was obtained as 44 (SD=7.8)/65 (SD=6.8) and 29 (SD=5.7)/62 (SD=7.8) months, respectively. The Kaplan-Meier survival analysis indicated significant differences between DSS (log-rank test; *P*=0.026) or PFS (log-rank test; *P*=0.005) and patients with tumors with high and low expression levels of talin-1 (Figures 6A and 6B). Moreover, the 5-year survival rate for the DSS or PFS of the patients with high talin-1 expression was 32% and 32%, and in those with low talin-1 expression, it was 63% and 59%, respectively (*P*=0.038 and *P*=0.002). 

**Fig. 7:**
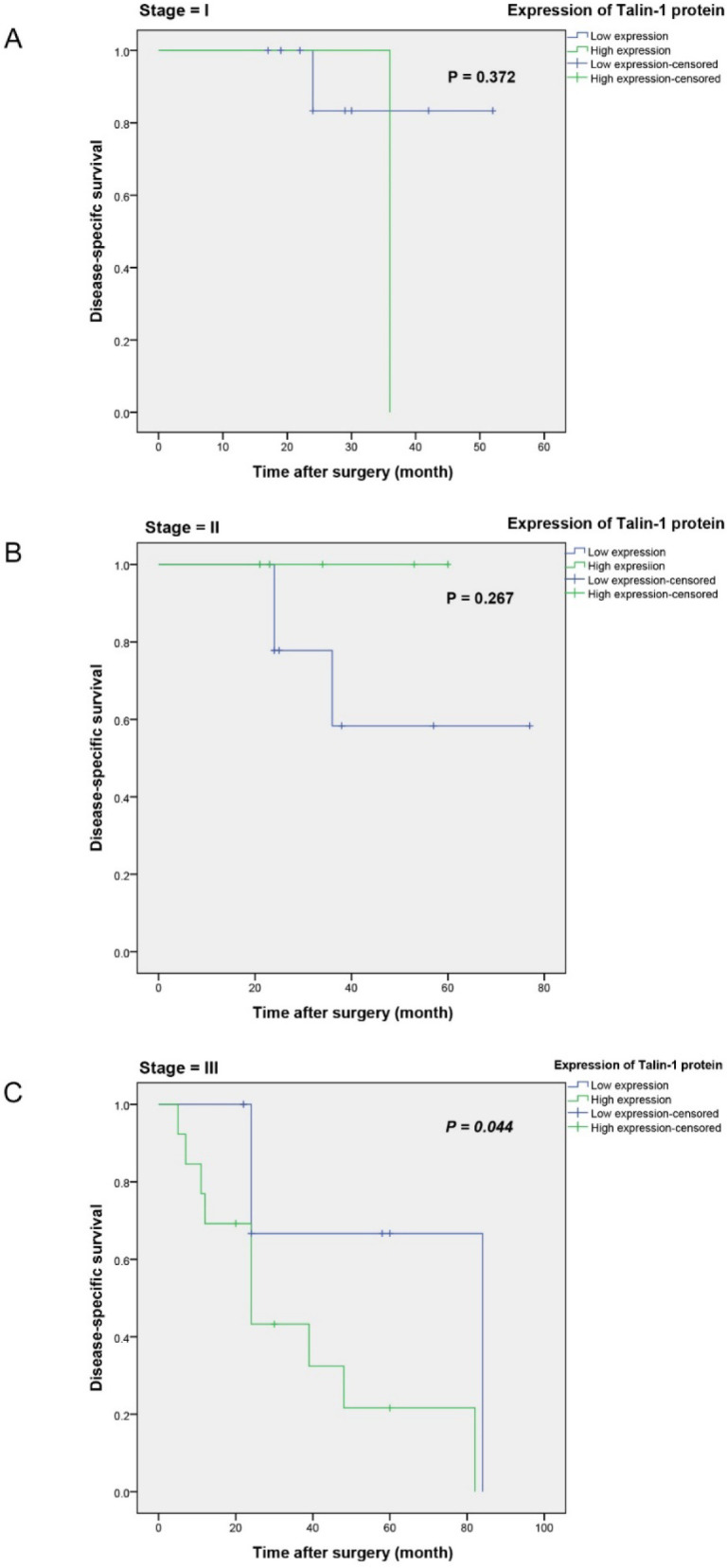
Disease-specific survival (DSS) curves for the patients at (A) FIGO stage I, (B) stage II, and (C) stage III of ovarian serous carcinoma (OSC). The results indicated that high expression of Talin-1 protein is associated with a poorer prognosis in the OSC patients with the advanced disease (FIGO stage III) (*P*=0.044).

The stratified analysis showed that in the subgroup of FIGO stages I and II (n=10 and n=15), there were no statistically significant differences in DSS between patients with high and low expression levels of talin-1 (*P*=0.26 and *P*=0.47; Figures 7A and 7B). However, in the subgroup of FIGO stage III (n=21), the patients with high talin-1 expression had significantly worsened DSS (*P*=0.04) compared to those with low talin-1 expression (Figure 7C).

**Fig. 8:**
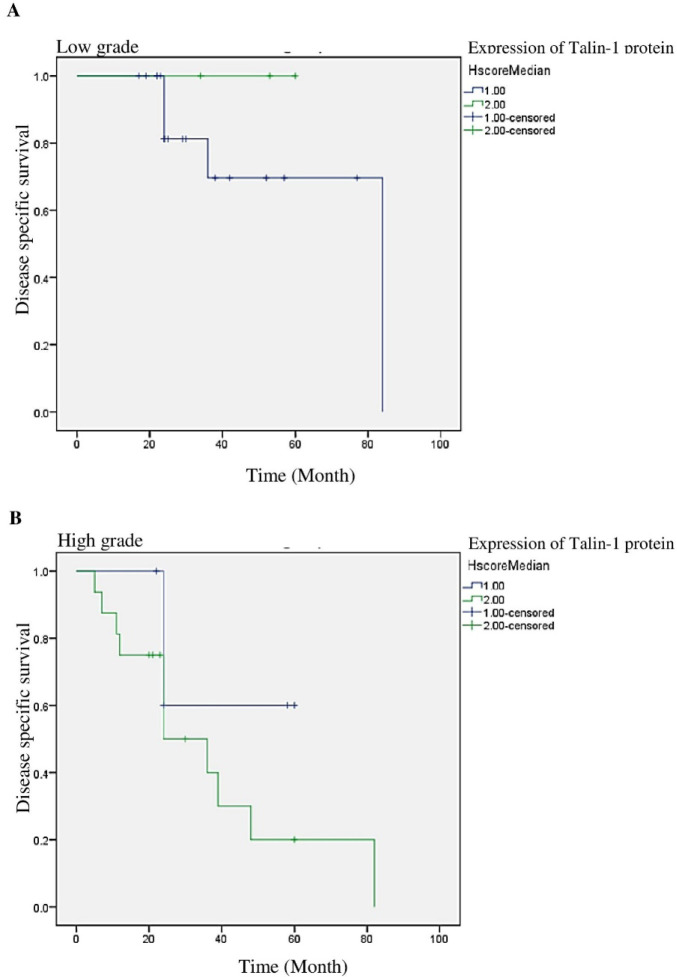
Disease-specific survival (DSS) curves for patients in (A) low-grade, (B) high-grade ovarian serous carcinoma (OSC). The results indicated that high level expression of Talin-1 is not significantly related to DSS in the OSC patients in high & low grades.

In addition, the stratified analysis exhibited that there was no significant association between the patients with high or low expression levels of talin-1 and high & low histological grades (Figures 8 A and 8B). Cox univariate and multivariate analyses were also used to assess the clinical significance of various parameters that might affect DSS or PFS in the OSC patients. As summarized in Table 4, the expression levels of talin-1 (hazard ratio [HR]: 2.791; 95% CI [1.029 to 1.571]; *P*=0.044) and histological grades (*P*=0.007), particularly high grade vs. low grade (HR: 4.101; 95% CI [0.910 to 11.603]; *P*=0.014), were significant risk factors, affecting the DSS of OSC patients in the univariate analysis. Some other variables, including omentum involvement (*P*=0.05), cervix involvement (*P*=0.002), myometrium involvement (*P*=0.012), post-cul-de-sac involvement (*P*=0.005), distant metastasis (*P*=0.001), and tumor recurrence (*P*=0.025) had *P *values less than 0.05; however, HR was not more than 1 (Table 4). Therefore, these variables were not included in the multivariate analysis. In addition, the expression levels of talin-1 (HR: 3.581; 95% CI [1.314 to 9.756]; *P*=0.013), histological grades (*P*=0.003), and FIGO stages (*P*=0.017) were known as the significant risk factors that could affect PFS in univariate analysis. Table 5 includes variables with significant P-values and low HR. However, the expression levels of talin-1 and FIGO stages were not significant risk factors affecting the DSS or PFS in multivariate analysis (Tables 4 and 5). Notably, the other clinicopathological variables were not significant factors for DSS or PFS in univariate and multivariate analyses of OSC patients.

**Table 4:** Univariate and multivariate Cox regression analyses of potential prognostic factor for disease-specific survival (DSS) in patients with ovarian serous carcinoma

Covariate	Univariate analysis		Multivariate analysis	
	HR (95% CI)	P-value	HR (95% CI)	P-value
Talin-1 expression High versus Low	2.791 (1.029- 7.571)	***0.044***	1.599 (0.500-5.108)	0.428
High grade versus low grade	4.101 (1.336 - 12.588)	***0.014***	3.250 (0.910- 11.603)	0.069
Omentum involvement	0.280 (0.075- 1.045)	***0.058***	-	-
Cervix involvement	0.179 (0.059- 0.538)	***0.002***	-	-
Myometrium involvement	0.245 (0.081- 0.737)	***0.012***	-	-
Post-cul-de-sac involvement	0.214 (0.073- 0.625)	***0.005***	-	-
Distant metastasis	0.089 (0.020- 0.395)	***0.001***	-	-
Tumor recurrence	0.011 (0.000- 0.564)	***0.025***	-	-

*H-score* histological score

Values in bold are statistically significant

The variables with *P* value less than 0.05 and HR more than 1.0 were included in multivariable analyses*HR *hazard ratio, *CI *confidence interval

**Table 5:** Univariate and multivariate Cox regression analyses of potential prognostic factor for progression-free survival (PFS) in patients with ovarian serous carcinoma

Covariate	Univariate analysis		Multivariate analysis	
	HR (95% CI)	P-value	HR (95% CI)	P-value
Talin-1 expression High versus Low	3.581 (1.314- 9.756)	***0.013***	1.905 (0.583- 6.226)	0.286
High grade versus low grade	5.460 (1.756- 16.976)	***0.003***	4.031 (1.155- 14.073)	***0.029***
FIGO stage		***0.017***		0.583
II versus I	0.846 (0.141- 5.069)	0.854	0.751 (0.123- 4.567)	0.756
III versus I	4.147 (0.926- 18.581)	0.063	1.672 (0.294- 9.511)	0.562
Vascular invasion (VI)	0.283 (0.094- 0.852)	***0.025***	-	-
Cervix involvement	0.179 (0.059- 0.538)	***0.002***	-	-
Myometrium involvement	0.245 (0.081- 0.737)	***0.012***	-	-
Vagina involvement	0.279 (0.097- 0.799)	***0.017***	-	-
Peritoneum involvement	0.257 (0.071- 0.931)	***0.039***	-	-
Colon involvement	0.303 (0.107- 0.855)	***0.024***	-	-
Post-cul-de-sac involvement	0.214 (0.073- 0.625)	***0.005***	-	-
Distant metastasis	0.089 (0.020- 0.395)	***0.001***	-	-
Tumor recurrence	0.011 (0.000- 0.564)	***0.025***	-	** -**

*H-score* histological score

Values in bold are statistically significant

The variables with P-value less than 0.05 and HR more than 1.0 were included in multivariable analysesHR hazard ratio, CI confidence interval

## Discussion

Despite some recent advances in the treatment of OC patients, this disease is still considered one of the deadliest cancers among gynecologic tumors (28). Therefore, the investigation of different markers and pathways for OC to recognize new markers for appropriate treatment is crucial. 

A primary search performed to investigate talin-1 expression as a prognostic biomarker using bioinformatics tools (29) demonstrated antithesis results in OC data. *In silico* analysis indicated both up- and downregulation in mRNA levels for talin-1 from OC tissues. The UALCAN data exhibited a significant decrease in the expression level of the talin-1 protein in OC tissues compared to normal tissues, while Wang *et al.* observed a high expression level of the talin-1 protein in OC patients (30). Additionally, to the best of our knowledge, the prognostic significance of talin-1 in OC patients remains largely unknown. Therefore, the present study was designed to evaluate the expression pattern, clinical significance, and prognostic value of talin-1 expression with various clinicopathological parameters by applying the IHC technique on TMA sections from OSC, benign ovarian tumors, and normal ovarian samples. Our results indicated that the expression of talin-1 was upregulated in the OSC patients than in benign tumors and normal tissue samples. Furthermore, in the current study, benign tumors showed higher expression of talin-1 compared to normal tissues. These findings are in line with previous studies on the OC patients, indicating that talin-1 expression significantly increases in the OC tissues, while its expression is weak or undetectable in benign and normal ovarian tissues (10, 11). Furthermore, these data are in agreement with several reports on the other carcinomas, including CRC (31), prostate cancer (32), nasopharyngeal carcinoma (13), HCC (9), and OSCC (12). 

Our results exhibited a significant association between increased talin-1 expression and higher histological grade and advanced FIGO stage. The median expression of talin-1 was found to be significantly higher in the OC patients with high histological grade and advanced FIGO stage compared to those with low histological grade and early FIGO stage. Accordingly, these data showed that the increased expression of talin-1 was related to the degree of malignancy and more advanced stages of disease in these cases. Furthermore, we found that histological grade and FIGO stage can be considered as prognostic factors in univariate analysis. Moreover, talin-1 expression added prognostic values of DSS and PFS in the OC patients in high grade vs. low grade; this shows that the expression of talin-1 is associated with the progression of disease in the OC patients. Previous studies have shown that higher histological grade and advanced tumor stage lead to poor clinical outcomes in patients with cancer (33-35). According to the definitive role of the talin-1 protein in integrin activation, which causes tumor progression and metastasis [36], our finding exhibited that higher talin-1 expression was significantly associated with myometrium involvement, indicating the role of talin-1 in tumor invasiveness and progression of the disease. This finding is in agreement with Wang *et al.*, showing that talin-1 plays a role in promoting the invasion and migration of OC (11). Therefore, our results indicated that the increased expression of talin-1 was related to the expansion of the disease and potential invasiveness.

Our study, for the first time, demonstrated that the OC patients with high expression of talin-1 had a worse prognosis for both DSS and PFS compared with those with low expression of talin-1. Likewise, the patients with higher levels of talin-1 showed a shorter 5-year survival rate for DSS or PFS compared with those with lower levels of expression. In addition, our stratified analysis showed that the OSC patients with high talin-1 protein expression in FIGO stage III had a significantly worse prognosis for DSS compared to those with low talin-1 protein expression. Altogether, these data indicated that high talin-1 protein expression was associated with poorer prognosis in the OSC, particularly in patients in the advanced stage. This finding is in line with a previous study on nasopharyngeal carcinoma, indicating that high expression of talin-1 is associated with a significantly worse survival rate in patients in stage III-IV of the disease (13). The expression of the talin-1 protein was recognized as a significant risk factor affecting the DSS or PFS in univariate analysis––but not in the multivariate analysis. Given the small number of cancer-related deaths or events, a longer follow-up time is necessary to determine the prognostic value of talin-1 expression. Increasing the follow-up period may reveal a stronger association between talin-1 expression and prognosis.

The results of several previous studies on other cancers are in line with our findings, indicating that upregulation of talin-1 is significantly associated with cancer progression and worse prognosis. Overexpression of talin-1 was found to be associated with more advanced clinicopathological characteristics, diminished survival rates, and the prediction of lymph node metastases among patients with prostate cancer - (36). A study on colon cancer demonstrated a significant correlation between increased expression of talin-1 and tumor grade, The TNM Classification of Malignant Tumors (TNM) stage, and lymph node metastasis (37). - In addition, the results of talin-1 protein expression on the CRC tissue samples in comparison to adjacent normal tissues revealed that increased expression of talin-1 protein in cancer tissues rather than adjacent normal tissues therefore the knockdown of talin-1 may reduce the proliferation and migration of the cancer cells (31). These findings suggested that the talin-1 protein is the main molecule involved in the progression of cancer. 

Talin-1 has shown paradoxical expression at the protein level in other malignancies and cancer research. Our previous study demonstrated that low-level expression of talin-1 was associated with advanced pathological features and poor DSS in the CRC patients (14). Moreover, HCC studies have found both up- and downregulation of talin-1 in tumor tissues (9, 15). Notably, talin was found to be completely absent in endometriosis and endometrioid carcinomas tissues (16). To date, the mechanisms that lead to the high and low expression levels of talin-1 in various cancers are not very clear. In this regard, further studies are needed to elucidate these mechanisms and better understand the role of talin-1 in cancer. By identifying the exact mechanism of talin-1, novel targeted therapies could be developed using this molecule. Moreover, a larger sample size and longer follow-up could help to improve our study regarding the prognostic impact of talin-1 in OSC.

## Conclusion

The dysregulation of talin-1 expression existed in tumor tissues from OSC patients. Moreover, using the IHC method, the upregulation of the talin-1 protein was significantly detected in OSC tumors than in benign tumors and normal tissues. Also, talin-1 protein expression was associated with more aggressive tumor behavior, more advanced disease, and poor DSS, especially in patients in the advanced stage of the disease (stage III) or in OSC patients with poor PFS. Therefore, talin-1 may have the potential as a novel poor prognostic biomarker of cancer-related death and progression of the disease in OC patients. However, further research is needed to fully understand the role of talin-1 in tumors.

## Funding

None.

## Conflict of Interest

The authors declare that they have no conflict of interest.
